# 3D-Printed Magnetic Polysaccharide Hydrogels as Sustainable Platforms for Pesticide Remediation

**DOI:** 10.1021/acsomega.6c03353

**Published:** 2026-08-07

**Authors:** Yulianis P. Barragán Medina, Jéssica S. Rodrigues, Amanda S. M. de Freitas, Gabriela P. U. Villarreal, Jimena S. Gonzalez, Leonardo F. Fraceto

**Affiliations:** † Grupo de Materiales Compuestos Termoplásticos, Instituto de Investigaciones en Ciencia y Tecnología de Materiales (INTEMA), CONICET-UNMdP, Av. Colón 10850, 7600 Mar del Plata, BA, Argentina; ‡ Institute of Science and Technology, São Paulo State University (UNESP), Av. Três de Março 511, 18087-180 Sorocaba, SP, Brazil

## Abstract

Magnetic hydrogels based on gum arabic (GA) and sodium alginate (SA) were fabricated by three-dimensional (3D) printing and functionalized with GA–stabilized magnetic iron oxides nanoparticles (GA-MNP) for the removal of the neonicotinoid pesticide imidacloprid from aqueous media. The nanoparticles (NP) preserved their crystalline magnetite structure after printing and calcium-mediated cross-linking, as confirmed by Fourier transform infrared spectroscopy (FTIR), X-ray diffraction (XRD), transmission electron microscopy (TEM), and thermogravimetric analysis (TGA). The printed hydrogels exhibited pH-responsive swelling behavior, with maximum hydration under acidic conditions. Adsorption experiments revealed that the formulation containing the highest NP loading achieved the greatest removal efficiency, reaching an adsorption capacity of 38.46 mg·g^–1^. Kinetic data were best described by the pseudo-second-order model, and equilibrium results followed the Langmuir isotherm, indicating monolayer adsorption governed by surface interactions. Overall, the combination of natural polymers, magnetic NP, and additive manufacturing provides a sustainable, efficient, and magnetically recoverable platform for the treatment of pesticide-contaminated water.

## Graphical abstract

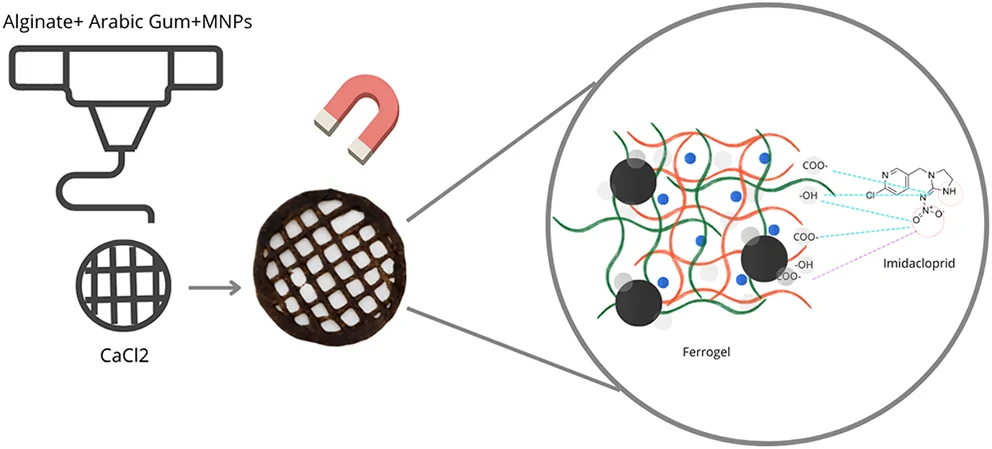

## 1 Introduction

Emerging contaminants constitute a diverse class of chemical substances that, despite the absence of comprehensive regulation, have raised significant environmental concern due to their persistence, bioactivity, and potential ecological risks.^1^ Among these contaminants, imidacloprid, a widely used neonicotinoid pesticide, has received particular attention due to its classification as a possible carcinogen by the European Food Safety Authority (EFSA) and its widespread distribution in aquatic environments.^2^ Its high solubility, chemical stability, and resistance to biodegradation facilitate its transport throughout the hydrological cycle, leading to its detection in surface waters, groundwater, agricultural runoff, and wastewater. These properties contribute to its documented toxicity toward nontarget organisms, including aquatic invertebrates and pollinators, thereby disrupting trophic dynamics and exacerbating global environmental pressures.^3^

Given these concerns, substantial efforts have been devoted to developing effective remediation strategies for imidacloprid removal. Adsorptive materials have emerged as a versatile and efficient class of solutions, including chitosan-based membranes functionalized with silver nanoparticles (NP), which improve sorption performance through electron-sharing interactions at the adsorbent–adsorbate interface.^4^ Similarly, biocarbon produced from water hyacinth and comodified with Fe/Mg has demonstrated a high affinity for imidacloprid via mechanisms including hydrogen bonding, π–π interactions, electrostatic interactions, and surface complexation.^5^

In parallel, electrochemical advanced oxidation processes (EAOPs) have been explored for the mineralization of imidacloprid, with the Electro-Fenton (EF) and Photo-Electro-Fenton processes demonstrating significant degradation efficiency through the generation of hydroxyl radicals (^•^OH) triggered by Fe^2+^-mediated activation of H_2_O_2_ under UV–C irradiation.^6^ Despite their effectiveness, such techniques often involve elevated operational costs, require strict pH control, and may generate toxic intermediates, such as Of-IMI and 5-OH-IMI, which can exhibit even higher acute and chronic toxicity to pollinators and aquatic organisms.^7^

These limitations highlight the need for sustainable adsorptive systems. Hydrogel-based materials, particularly those derived from natural polymers, have attracted increasing attention due to their tunable porosity and functional chemistry. Alginate hydrogels are widely used in environmental and agricultural applications; however, most systems are limited to non-3D configurations and are primarily designed for controlled release rather than adsorption, such as alginate-based formulations for imidacloprid delivery.^8^ For example, alginate/chitosan composite hydrogels have been reported as pH-responsive carriers for controlled pesticide release,^8^ whereas other alginate systems rely on diffusion-controlled release mechanisms, which limit their applicability in pollutant-removal scenarios.^9^

Gum Arabic (GA) is an attractive biopolymer for fabricating sorptive hydrogels due to its natural abundance, biodegradability, and diverse functional groups (−OH, −COOH, −NH_2_), which promote hydrogen bonding, electrostatic interactions, and coordination with contaminants.^10,11^ Importantly, previous studies have demonstrated that GA can significantly improve the rheological behavior, viscosity, and mechanical stability of polysaccharide-based systems, which are critical parameters for extrusion-based 3D printing. In addition, GA can act as a stabilizing agent for metal oxide NP, enhancing their dispersion and preventing aggregation. In this work, GA plays a dual and synergistic role: it enhances the printability and structural fidelity of the hydrogel, enabling the fabrication of well-defined 3D architectures, and it contributes to adsorption performance by increasing the density of functional groups and promoting a more homogeneous and accessible porous network, thereby facilitating mass transfer and pollutant–matrix interactions.^12,13^

3D printing has recently emerged as a transformative platform for manufacturing hydrogel-based adsorbents with tailored geometry, controlled mechanical properties, and tunable internal architectures.^10,11^ Layer-by-layer deposition enables the fabrication of networks with optimized porosity and diffusion pathways, thereby improving contaminant-sorbent interactions, reducing fouling, and facilitating post-treatment handling.^14^ Despite recent advances in 3D-printed magnetic hydrogels, most reported systems rely on alginate–methylcellulose blends or synthetic polymers and are primarily focused on biomedical or actuation-related applications rather than environmental remediation.^10,11^ Although GA–SA composite materials have previously been investigated for adsorption applications, particularly for the removal of heavy metals, their integration into a 3D-printed magnetic hydrogel architecture incorporating gum Arabic-stabilized iron oxide nanoparticles for the adsorption of organic contaminants such as imidacloprid remains largely unexplored. This combination represents a relevant research opportunity at the intersection of sustainable biomaterials, additive manufacturing, and water remediation technologies.

Given this context, the present work proposes the development of 3D-printed magnetic hydrogels based on GA and SA, functionalized with GA-stabilized iron oxide NP, for the efficient removal of imidacloprid from aqueous media. To achieve this, magnetic NP were synthesized and thoroughly characterized, followed by the formulation of printable biopolymeric inks, the fabrication of 3D hydrogel architectures, and the evaluation of their physicochemical properties, swelling behavior, and adsorption performance. This integrated strategy, combining natural polymers, magnetic nanostructures, and additive manufacturing, establishes a novel platform for the design of sustainable and efficient adsorbent materials targeting persistent agricultural contaminants.

## 2 Materials and Methods

### 2.1 Materials

GA and SA were purchased from Sigma-Aldrich (Brazil and the United Kingdom, respectively). Ferric chloride hexahydrate (FeCl_3_·6H_2_O; *M*_p_ = 270.30 g·mol^–1^) was obtained from Neon, Brazil, and ferrous sulfate heptahydrate (FeSO_4_·7H_2_O; *M*_p_ = 278.01 g·mol^–1^) from Labsynth Ltd.a, Brazil. Calcium chloride dihydrate (CaCl_2_·2H_2_O; *M*_p_ = 147.01 g·mol^–1^) and sodium hydroxide (NaOH; *M*_p_ = 40.00 g·mol^–1^) were both acquired from Dinâmica Ltd.a, Brazil.

### 2.2 Synthesis of GA-Based Iron Oxide Nanoparticles

Magnetic nanoparticles (MNP) were synthesized (Figure 1) via coprecipitation in the presence of GA as a stabilizing agent. Briefly, a 0.5% (w/v) aqueous GA solution was prepared under magnetic stirring at 60 °C. Subsequently, Fe^3+^ and Fe^2+^ were added at final concentrations of 1% and 0.5% (w/v), respectively, maintaining an Fe^3+^/Fe^2+^ molar ratio of 2:1. The mixture was stirred for 60 min, after which 21 mL of NaOH solution (1 M) was added dropwise until the pH reached 11, promoting the coprecipitation of GA-stabilized MNP (GA-MNP). The suspension was stirred for an additional 30 min, and the resulting GA-MNP were centrifugated at 1200 rpm to accelerate the separation process and reduce the time required for nanoparticle recovery. The supernatant was discarded, and the precipitate was washed five times with ultrapure water, using a neodymium magnet to facilitate separation. The final GA-MNP suspension contained 4% (w/w). The GA-MNP suspension was stored at room temperature (24 ± 1 °C) and utilized shortly after synthesis. Under these conditions, the gum Arabic coating provided sufficient steric stabilization to prevent noticeable aggregation, and no changes in dispersion stability or magnetic response were observed during storage. Therefore, refrigeration was not required for the duration of the experiments.

**Figure 1:**
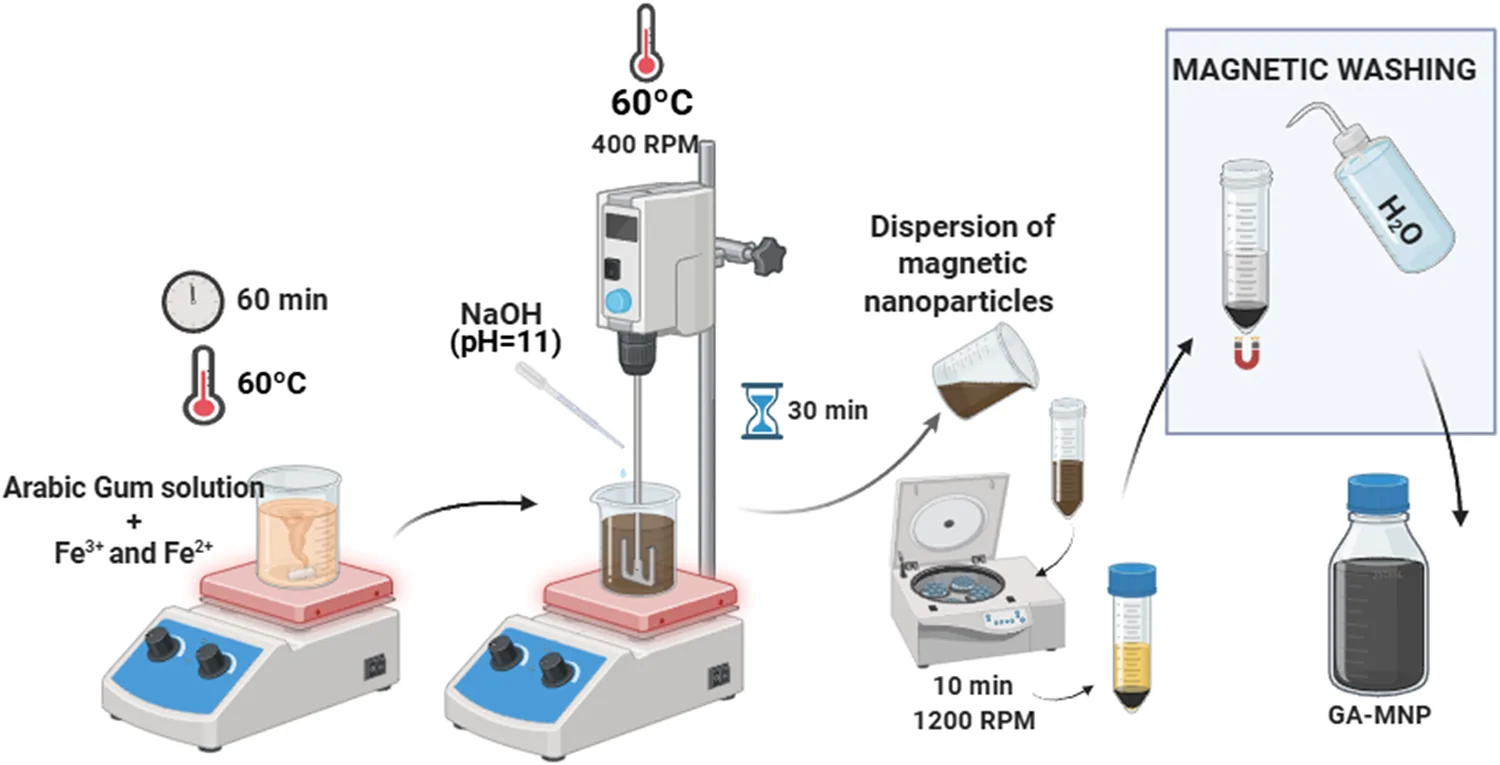
Schematic illustration of the synthesis of gum arabic–stabilized magnetic nanoparticles (GA-MNP) by coprecipitation. An aqueous gum arabic solution containing Fe^3+^ and Fe^2+^ ions (molar ratio 2:1) is heated and magnetically stirred, followed by alkaline coprecipitation induced by NaOH addition (pH ≈ 11), leading to the formation of magnetite nanoparticles stabilized by the biopolymer matrix. The resulting suspension is centrifuged and subjected to successive magnetic washing steps to remove unreacted species and loosely bound components, yielding purified GA-MNP dispersions that preserve magnetic responsiveness and colloidal stability.

### 2.3 Formulation and Printing of Biopolymeric Inks

Four distinct biopolymeric inks were prepared to evaluate both printability and adsorption performance (Figure 2). All formulations were prepared at 60 °C by dispersing GA and SA in ultrapure water, followed by incorporation of GA-MNP when applicable. Initially, two formulations with different viscosities were designed (L_GA_-MNP_10_ and H_GA_-MNP_10_) by varying the GA/SA ratio while keeping the GA-MNP content constant at 10% (v/v) to decouple the effect of polymer composition from nanoparticle loading

**Figure 2:**
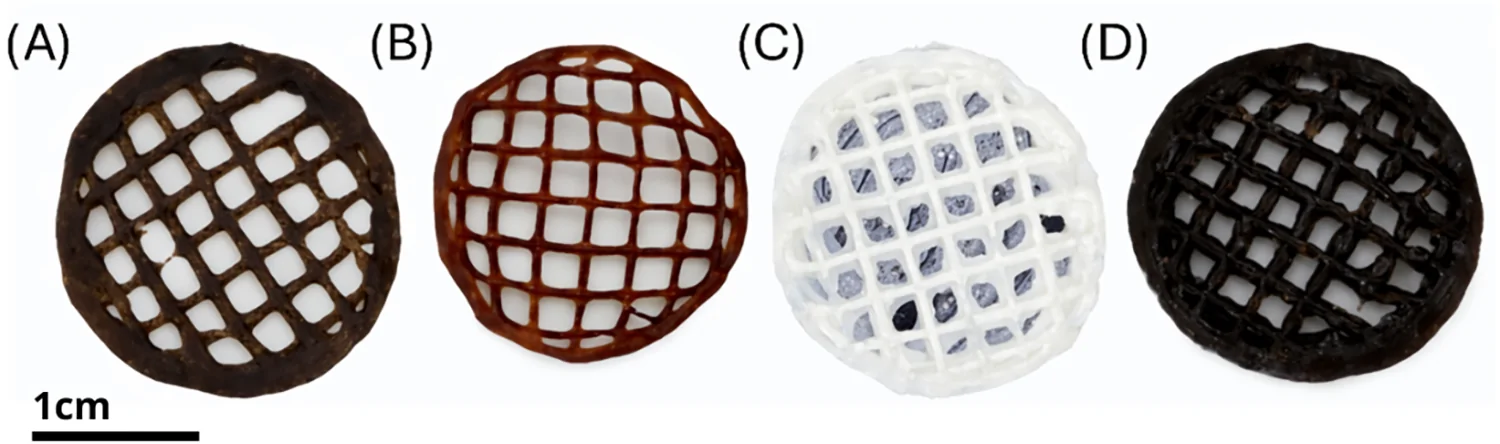
Digital photographs of 3D-printed GA/SA-based hydrogels: (A) L_GA_-MNP_10_, (B) H_GA_-MNP_10_, (C) H_GA_-Ctrl, and (D) H_GA_-MNP_20_.

L_GA_-MNP_10_, with a low GA/SA ratio (0.04, w/w), exhibited a markedly high viscosity due to the higher relative alginate content, which promotes stronger chain entanglement and intermolecular interactions within the polymeric network. This hindered continuous extrusion, leading to irregular filament deposition and poor shape fidelity. In contrast, increasing the GA/SA ratio to 0.50 (w/w) in H_GA_-MNP_10_ reduced viscosity to a range compatible with extrusion-based printing, enabling stable filament formation, improved flow through the nozzle, and enhanced structural integrity of the printed constructs. This behavior is consistent with the reduction in intermolecular interactions within the polymer network at higher GA content, facilitating shear thinning and flow under applied stress.

Based on these observations, the GA/SA ratio of H_GA_-MNP_10_ was selected as the optimal balance between viscosity and printability. This composition was subsequently used as a baseline to isolate the effect of nanoparticle loading. Accordingly, a control formulation without nanoparticles (HGA-Ctrl) and a higher loading system (H_GA_-MNP_20_, 20% v/v; 0.8% w/w solids) were prepared to evaluate the influence of GA-MNP concentration on adsorption performance while maintaining comparable rheological behavior and printability.

The compositions of all inks are provided in Table S1. Each ink was loaded into 5 mL syringes and printed at 25 °C using a 0.7 mm nozzle. The main printing parameters were as follows: print speed of 6 mm·s^–1^, layer height of 0.3 mm, infill pattern of grid, and infill density of 3%. Printability was assessed based on extrusion continuity, filament homogeneity, strand deposition, and structural fidelity after printing.

After printing, the constructs were immersed in a 2% (w/v) CaCl_2_ solution for ionic cross-linking and hydrogel formation, followed by two rinses in ultrapure water to remove unbound Ca^2+^. The printing procedure was adapted from the methodology reported by Villarreal et al.,^15^ using a Génesis bioprinter (3D Biotechnology Solutions). The three-dimensional model was developed in the 3D Printer Slicer v5.0.3 software and printing execution in the Pronterface v3 software, employing the microextrusion method.

### 2.4 Physicochemical and Structural Characterization

#### 2.4.1 Physicochemical Characterization of GA-MNP

Nanoparticle size distribution and concentration were analyzed by nanoparticle tracking analysis (NTA) using an NS300 system (Malvern Instruments, U.K.) equipped with a 488 nm laser, recording five 60 s videos per sample and using the viscosity of water at 25 °C. The crystalline phase was identified by X-ray diffraction (XRD) using a Bruker D8 Advance diffractometer with Cu Kα radiation (λ = 1.5418 Å), operating at 40 kV and 40 mA, in the 2θ range of 10–80° with a 0.02° step size. Chemical structures were assessed by Fourier transform infrared spectroscopy (FTIR, Agilent) in attenuated total reflectance (ATR) mode, averaging 32 scans at 4 cm^–1^ resolution over the range 4000–400 cm^–1^. Morphology and particle size were further examined by Transmission Electron Microscopy (TEM) using a JEOL 100 CX, with images acquired at magnifications of 200,000× and 370,000×.

#### 2.4.2 Structural, Morphological, and Functional Characterization of Hydrogels

Thermal properties were evaluated by thermogravimetric analysis (TGA, TA Instruments Q50) under nitrogen (20 mL·min^–1^) from 25 to 900 °C at 20 °C·min^–1^, and by differential scanning calorimetry (DSC, TA Instruments Q2000) under nitrogen (50 mL·min^–1^) from 0 to 300 °C at 20 °C·min^–1^. The crystalline structure was analyzed by XRD. The hydrogel density was determined by liquid pycnometry using distilled water as the immersion medium at constant temperature (23 °C). The mass of the empty pycnometer (*m*_0_) and the mass of the pycnometer filled with water (*m*_w_) were first recorded to calibrate the effective volume. A known mass of dry hydrogel (*m*_s_) was then introduced into the dry pycnometer, which was subsequently filled with distilled water to the calibration mark. The mass of the system (*m*_sw_) was measured after ensuring the absence of trapped air bubbles. The hydrogel density (ρ_s_) was calculated as ρ_s_=(*m*_s_/*m*_s_ + *m*_w_ – *m*_sw_)* ρ_w_, where ρ_w_ is the density of water at the experimental temperature. Atomic force microscopy (AFM) analysis of the hydrogels was performed using an Easyscan 2 microscope (Nanosurf, Switzerland) operated in noncontact mode with TapAl-G cantilevers (BudgetSensors, Bulgaria) at a scan rate of 90 Hz. Surface and morphology images were processed and analyzed using Gwyddion v2.0 software. The chemical structures of the hydrogels before and after adsorption experiments were analyzed by FTIR, as described for GA-MNP.

### 2.5 Swelling Behavior

The swelling behavior of the hydrogels was evaluated gravimetrically. Adsorption experiments were performed at pH 3, 7, and 9, (adjusted with HCl or NaOH solutions), and room temperature (24 ± 1 °C), throughout the experiments. Room-temperature-dried samples of known initial mass (*W*_0_) were immersed in aqueous solutions with controlled pH values 3, 7, and 9 and then maintained at 24 ± 1 °C. The samples were periodically removed from the swelling medium, gently blotted with filter paper to eliminate excess surface liquid, and weighed until a constant mass was reached (*W*_t_), indicating that swelling equilibrium had been achieved. The swelling ratio (SR) was calculated according to eq ([Disp-formula eq1]). All measurements were performed in triplicate for each pH condition.

1 $$\mathrm{SR}\;\left(\%\right)=\left(\left(W_{t}-W_{o}\right)/W_{o}\right)\times 100$$

### 2.6 Adsorption Assays for Imidacloprid Removal

Batch adsorption experiments were carried out by immersing hydrogels of known mass in aqueous imidacloprid solutions of predetermined initial concentration (5, 10, 15, 20 mg/L). For each adsorption experiment, 20 mg of hydrogel were immersed in 50 mL of IMI solution, corresponding to an adsorbent dosage of 0.4 g L^–1^. The suspensions were stirred continuously at room temperature (24 ± 1 °C). At predetermined time intervals, aliquots were withdrawn, filtered, and analyzed by UV–Vis spectrophotometry at 270 nm (λ_max)^5^ to determine the residual imidacloprid concentration. The equilibrium adsorption capacity (*q*_e_) was calculated according to eq 2.

2 $$q_{e}=\left(\left(C_{o}-C_{e}\right)\times V\right)/m$$

where *C*_0_ and *C*_e_ (mg·L^–1^) are the initial and equilibrium concentrations, respectively, *V* (L) is the solution volume, and *m* (g) is the mass of hydrogel.

### 2.7 Reuse Assays

To evaluate the reusability of the 3D-printed hydrogels, the samples were immersed in 50 mL of imidacloprid (IMI) solution at a concentration of 15 mg/L and maintained under constant stirring for 48 h. After the adsorption step, the hydrogels were removed from the solution, superficially rinsed with distilled water to eliminate residual IMI solution, and immediately transferred to 50 mL of desorption medium. Two desorption agents were evaluated for IMI regeneration: 0.5 M KCl for 48 h and 70% ethanol for 2 h. Following desorption, the hydrogels were again superficially rinsed with distilled water and directly reused in the subsequent adsorption cycle without any drying step. This adsorption–desorption procedure was repeated until a significant loss of adsorption capacity was observed. The recovery efficiency was calculated according to eq 3.

3 $$R\%=\left(\frac{q_{e}\;\mathrm{cycle}}{q_{e}\;\mathrm{first}\;\mathrm{cycle}}\right)\times 100$$

## 3 Results and Discussion

### 3.1 Morphological, Structural, and Surface Characterization of Iron Oxide Nanoparticles

The crystalline structure of the GA-MNP was confirmed by XRD (Figure 3A). The diffraction peaks at 2θ = 30.25°, 36.22°, 43.50°, 54.10°, 57.20°, 63.10°, and 75.00° correspond to the (220), (311), (400), (422), (511), (440), and (533) crystallographic planes of magnetite (Fe_3_O_4_), in agreement with the standard JCPDS card No. 19–0629. No additional reflections associated with hematite or maghemite were detected, indicating high crystallinity and phase purity of the NP obtained by coprecipitation.

**Figure 3:**
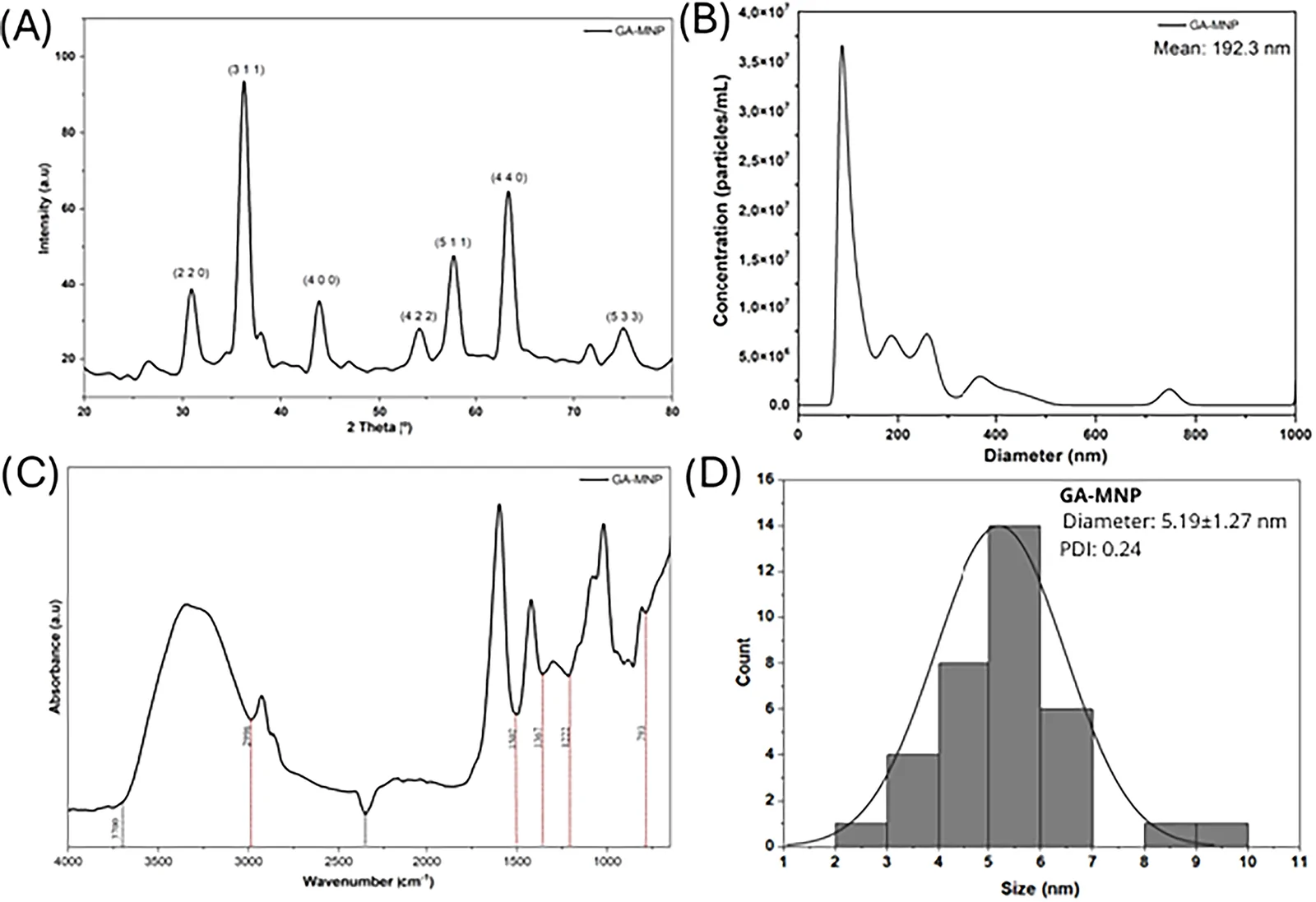
Physicochemical characterization of gum arabic–stabilized magnetic nanoparticles (GA-MNP). (A) X-ray diffraction (XRD) pattern showing diffraction peaks indexed to the characteristic crystallographic planes of magnetite (Fe_3_O_4_), confirming the formation of a highly crystalline magnetic phase. (B) Nanoparticle tracking analysis (NTA) revealing the hydrodynamic size distribution of GA-MNP in aqueous suspension, with a mean diameter of 192.3 nm, reflecting the polysaccharide coating and nanoparticle clustering in solution. (C) Fourier-transform infrared (FTIR) spectrum highlighting the characteristic vibrational bands of gum arabic, including O–H stretching, C–H stretching, and COO^–^-related modes, together with the Fe–O stretching band, confirming the successful stabilization of magnetite nanoparticles by the biopolymer. (D) Particle size distribution histogram obtained from transmission electron microscopy (TEM) images, showing nanometric, nearly spherical primary magnetite nanoparticles and confirming the formation of well-defined nanocrystalline domains.

The average crystallite size, estimated from the (311) reflection using the Scherrer equation, was approximately 3.6 nm. This value is significantly smaller than the hydrodynamic diameter measured by NTA, which showed a size distribution centered at 192.3 nm (Figure 3B). The discrepancy between the crystallite size obtained from XRD (∼3.6 nm) and the hydrodynamic diameter measured by NTA (∼192 nm) arises from the fundamentally different nature of these techniques. While XRD probes the size of coherent crystalline domains within individual MNP, NTA measures the hydrodynamic behavior of particles in suspension, encompassing not only the inorganic core but also the surrounding gum arabic shell and any nanoparticle aggregates present in the colloidal system.^16,17^

In aqueous media, MNP tends to undergo partial aggregation driven by dipole–dipole magnetic interactions. Although GA provides steric stabilization through its branched polysaccharide structure, preventing uncontrolled agglomeration, it does not completely suppress the formation of small clusters. This behavior is reflected in the multimodal NTA distribution, which exhibits a dominant population below 200 nm together with additional populations at larger diameters that can be attributed to nanoparticle aggregates. Consequently, the measured hydrodynamic diameter reflects a heterogeneous population rather than isolated primary particles. This aggregation behavior has important implications for the dispersion of NP within the printed hydrogel network. During ink formulation, the polymeric matrix (GA/SA) interacts with the GA-coated NP, promoting their distribution while preserving some degree of clustered organization. These NP clusters can act as localized reinforcing domains within the hydrogel, contributing to network structuring, mechanical stability, and the formation of heterogeneous adsorption sites. Therefore, the multiscale organization, comprising nanometric crystalline domains assembled into submicrometric aggregates, plays a key role in defining both the structural and functional performance of the 3D-printed ferrogels.

FTIR (Figure 3C) confirmed the effective stabilization of the magnetic core by GA. The broad band between 2996–3700 cm^–1^ was assigned to O–H stretching vibrations, while bands at 1222, 1367, and 1502 cm^–1^ correspond to C=O-related vibrational modes characteristic of GA. The presence of a distinct Fe–O stretching band at 793 cm^–1^ confirms the coexistence of the organic coating and the magnetite core, supporting the formation of a core–shell-like structure that provides steric stabilization and surface functionality for adsorption.^18,19^

TEM analysis (Figures S1 and 3D) corroborated the nanometric dimensions and morphology of the GA-MNP. Nearly spherical primary nanoparticles were observed, forming clusters consistent with the magnetic interactions of magnetite. To avoid overestimation caused by aggregation during sample drying, the particle size distribution was determined exclusively from isolated nanoparticles. The resulting average particle diameter of 5.19 ± 1.27 nm corresponds to the size of individual nanoparticles directly observed by TEM and confirms the formation of well-defined nanocrystalline nuclei. This value is consistent with the crystallite size estimated from XRD, whereas the substantially larger size measured by NTA reflects the hydrodynamic diameter of nanoparticle agglomerates dispersed in suspension, including the gum arabic coating and associated solvation layer. The slightly larger particle size observed by TEM compared to the crystallite size estimated by the Scherrer equation may be attributed to the presence of polycrystalline nanoparticles and/or to the inherent limitations of the Scherrer approach, which can underestimate crystallite size due to additional contributions to peak broadening such as microstrain and lattice defects.

Overall, the combined XRD, TEM, NTA, and FTIR results demonstrate a hierarchical organization of the GA-MNP system, consisting of nanocrystalline magnetite cores assembled into larger hydrodynamic entities stabilized by a gum arabic polymer layer. This multiscale structure ensures colloidal stability in aqueous media while preserving a high density of accessible surface functional groups, a key feature for efficient interaction with organic contaminants in adsorption-based water treatment applications.^20^

### 3.2 Morphological, Structural, and Thermal Characterization of 3D-Printed Ferrogels

The chemical, structural, and thermal characteristics of the 3D-printed ferrogels were investigated to elucidate the effect of GA-MNP incorporation and polymer composition on network integrity and stability. FTIR spectra of the printed ferrogels (Figure 4A) exhibited the characteristic −OH and C–H stretching bands at 3686 and 2970 cm^–1^, respectively, reflecting the hydrophilic nature of the GA/SA matrix. Vibrational bands at 1498, 1341, and 1215 cm^–1^ were assigned to COO^–^ and C–O–C groups from GA and SA, while the presence of a distinct Fe–O band at 786 cm^–1^ confirmed the successful incorporation of magnetite domains within the hydrogel structure. The main FTIR band assignments and representative band shifts observed between GA-MNP and the printed ferrogels are summarized in Table S2. Compared to the GA-MNP spectrum (Figure 3C), small but measurable shifts were observed, including the displacement of the Fe–O band from 793 to 786 cm^–1^ and minor variations in the COO^–^- and C–O-related bands. These spectral changes indicate coordination interactions between Fe_3_O_4_ surfaces and oxygenated functional groups (−OH and −COO^–^), as well as effective Ca^2+^-mediated ionic cross-linking within the polysaccharide network.^21^

**Figure 4:**
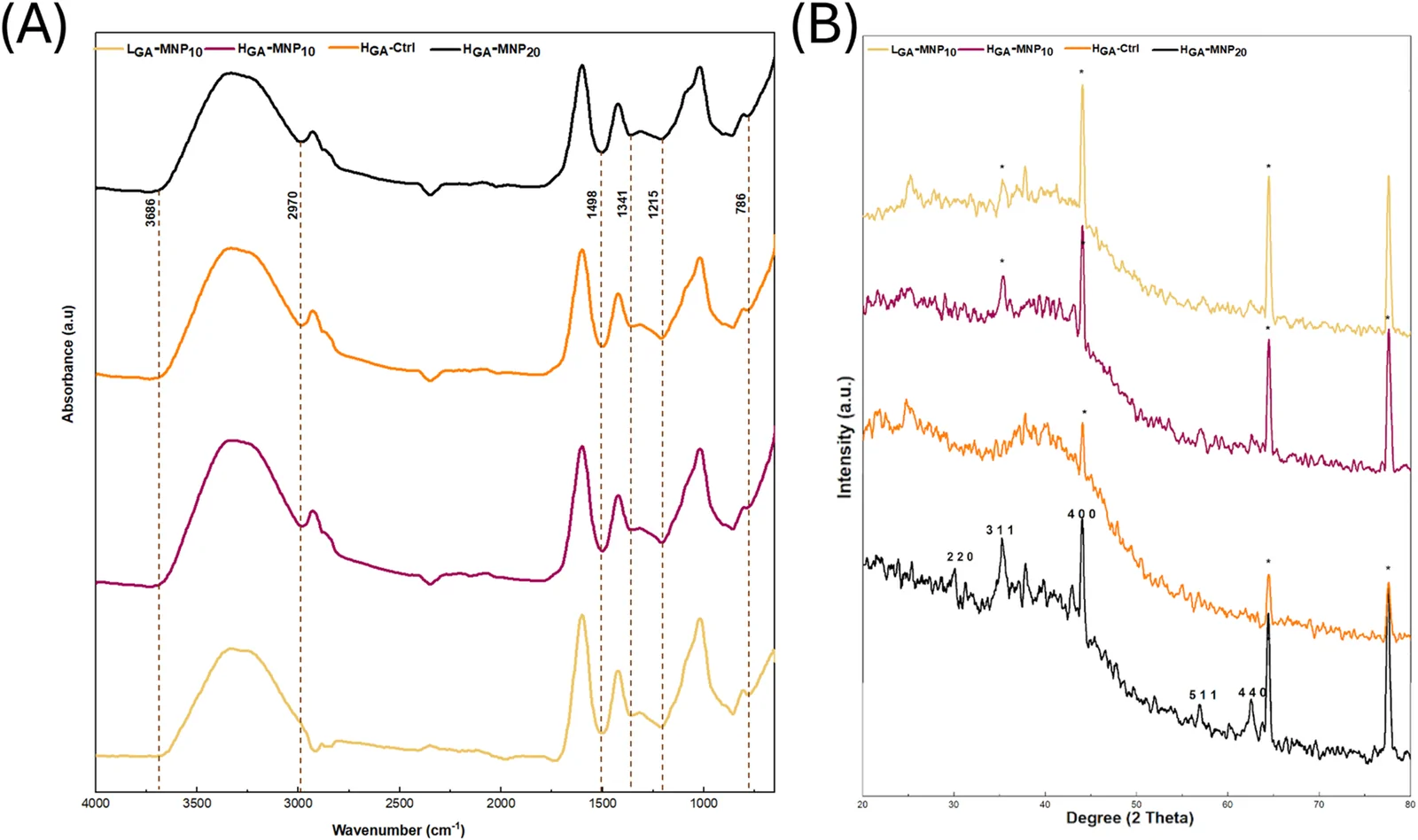
Chemical and crystalline characterization of 3D-printed GA/SA-based ferrogels with different magnetic nanoparticle loadings. (A) Fourier-transform infrared (FTIR) spectra of LGA-MNP10, HGA-MNP10, HGA-Ctrl, and HGA-MNP20 hydrogels, highlighting the characteristic O–H stretching band (∼3686 cm^–1^), C–H stretching (∼2970 cm^–1^), and polysaccharide-related COO^–^ and C–O–C vibrations (1498, 1341, and 1215 cm^–1^). The presence of the Fe–O stretching band (∼786 cm^–1^) confirms the successful incorporation of magnetite nanoparticles within the polymeric network. Dashed lines indicate key vibrational regions affected by nanoparticle content and polymer composition. (B) X-ray diffraction (XRD) patterns of the corresponding printed ferrogels, showing diffraction peaks indexed to the (220), (311), (400), (422), (511), and (440) crystallographic planes of magnetite (Fe_3_O_4_; JCPDS No. 19–0629). The progressive increase in peak intensity with higher GA-MNP content, particularly in HGA-MNP20, indicates increased magnetic phase incorporation, while the absence of secondary phases confirms the preservation of magnetite crystallinity after 3D printing and Ca^2+^-mediated ionic cross-linking.

XRD patterns (Figure 4B) exhibit diffraction peaks at 30.3°, 35.3°, 44.0°, 54.1°, 57.0°, 64.4°, and 75.5°, assigned to the (220), (311), (400), (422), (511), (440), and (533) planes of Fe_3_O_4_ (JCPDS 19–0629), confirming that the magnetite crystalline phase remains intact within the polymeric matrix.^22^ Peak intensity increases with MNP content, particularly in H_GA_-MNP_20_, as observed by FTIR. Using the Scherrer equation applied to the (311) reflection, the crystallite size remained ∼3.5 nm, like the pristine GA-MNPs. This demonstrates that 3D printing and ionic cross-linking did not induce particle growth or phase transformation, thereby preserving the crystalline integrity of the magnetite domains. Together, FTIR and XRD confirm both the chemical integration of GA-MNP via coordination to polysaccharide functional groups and the structural preservation of the crystalline Fe_3_O_4_ cores after 3D printing—an essential feature for maintaining magnetic responsiveness and adsorption efficiency.

These chemical and structural features are directly reflected in the surface organization observed by AFM (Figure S2). The L_GA_-MNP_10_ hydrogel exhibits a relatively homogeneous, gently undulated surface, indicating a balanced dispersion of nanoparticles within an alginate-rich matrix. Increasing the GA content at constant nanoparticle loading (H_GA_-MNP_10_) leads to a more structured surface with well-distributed reliefs, suggesting stronger polymer–nanoparticle interactions and localized network reorganization.^23^ In contrast, the nanoparticle-free HGA-Ctrl displays pronounced surface irregularities and poorly controlled topography, evidencing the absence of structuring domains capable of constraining polymer rearrangement. The highest nanoparticle loading (H_GA_-MNP_20_) produces the most compact and highly organized surface, characterized by well-defined reliefs, consistent with a denser and mechanically reinforced network.^24,25^

Thermal analysis further highlights formulation-dependent differences that directly reflect the structural organization inferred from FTIR and XRD. DSC curves (Figure 5A) show an initial endothermic event around 95–100 °C for all samples, attributed to the release of physically bound water associated with hydroxyl-rich polysaccharide chains. Beyond this dehydration step, distinct behaviors emerge. The thermal transitions observed between 200 and 250 °C are consistent with the degradation behavior reported for SA, which typically undergoes decarboxylation and backbone decomposition within this temperature range, whereas GA generally degrades between 200 and 300 °C depending on its molecular structure and branching degree. Therefore, the multiple transitions observed for H_GA_-Ctrl can be attributed to overlapping degradation events of the polysaccharide components. In contrast, GA-MNP–containing ferrogels show shifted and broadened transitions, indicating restricted polymer dynamics due to NP–polymer interactions. H_GA_-MNP_10_ presents two closely spaced transitions, reflecting the coexistence of polymer depolymerization and NP-related oxidative processes. H_GA_-MNP_20_ exhibits a further shift of these transitions to higher temperatures, consistent with increased interfacial interactions and a more compact structure that limits chain relaxation and delays thermal events. L_GA_-MNP_10_ exhibits intermediate behavior, with the alginate-rich composition contributing to thermal resistance despite the lower GA content.

**Figure 5:**
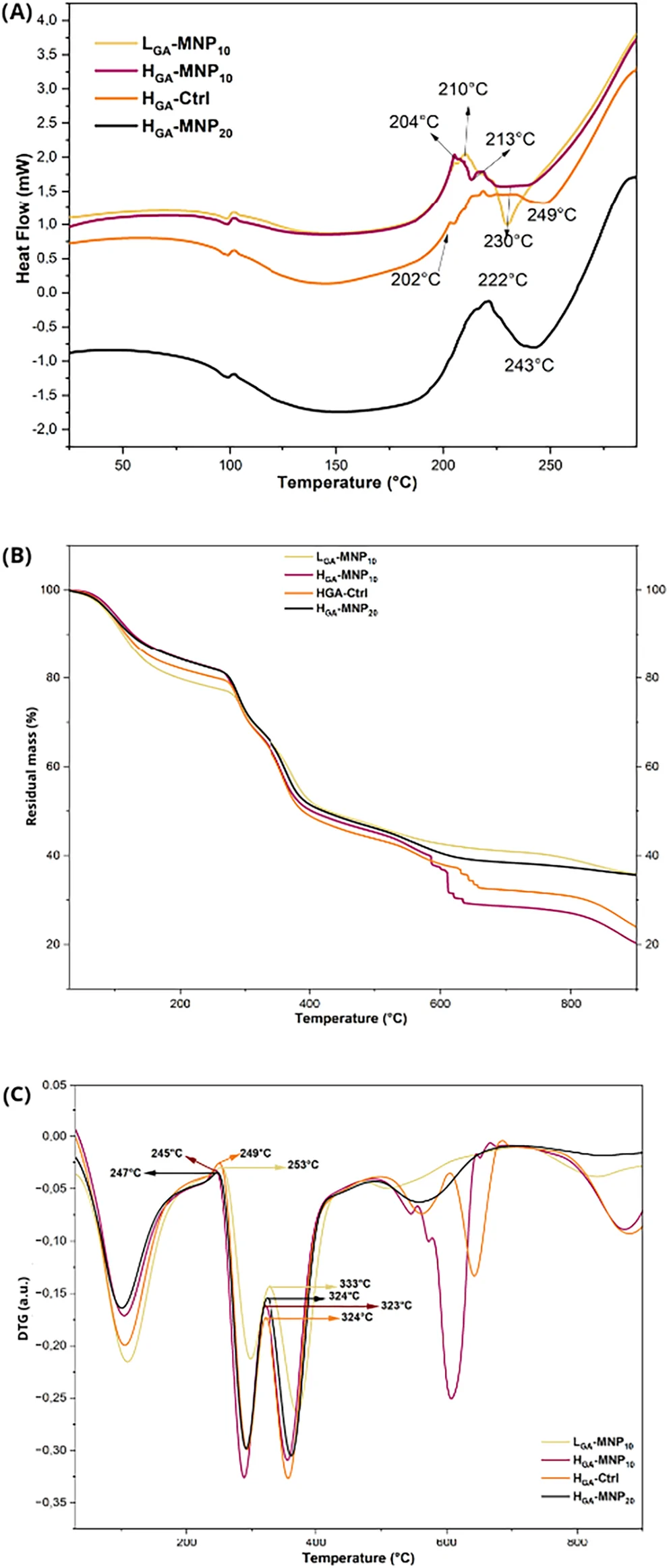
Thermal behavior and stability of 3D-printed GA/SA-based ferrogels with different magnetic nanoparticle loadings. (A) Differential scanning calorimetry (DSC) thermograms showing an initial endothermic event associated with the release of physically bound water, followed by formulation-dependent thermal transitions related to polymer chain relaxation and degradation. The progressive shift of thermal events toward higher temperatures with increasing GA-MNP content indicates restricted polymer mobility and enhanced thermal stability. (B) Thermogravimetric analysis (TGA) curves showing multistep mass-loss profiles, including initial dehydration and subsequent thermal degradation of the biopolymeric network. Ferrogels containing GA-MNP exhibit delayed degradation and higher residual mass compared to the nanoparticle-free control, reflecting the reinforcing effect of the magnetic phase. (C) Derivative thermogravimetric (DTG) curves highlighting distinct degradation stages and peak shifts as a function of polymer composition and nanoparticle loading, confirming the role of GA-MNP in increasing network compactness and thermal robustness of the printed hydrogels.

These trends are fully consistent with the TGA and DTG profiles (Figure 5B–C). All samples exhibit an initial mass loss of ∼160–200 °C due to the removal of free and bound water. The main degradation stages occur at higher temperatures and are strongly formulation dependent. H_GA_-Ctrl undergoes earlier degradation and leaves the lowest residual mass, confirming its reduced thermal robustness. In contrast, the GA-MNP-containing ferrogels exhibit improved thermal stability, with degradation events shifted toward higher temperatures. H_GA_-MNP_10_ shows enhanced thermal resistance relative to the control, while H_GA_-MNP_20_ presents the highest residual mass (∼36%) and the greatest delay in degradation events, indicating a more compact and thermally robust structure. However, this residue cannot be attributed exclusively to the magnetite content. Considering the formulation composition, the amount of incorporated NP is substantially lower than the final residue observed by TGA. Therefore, the residual mass likely results from the combined contribution of thermally stable Fe_3_O_4_ domains, calcium-containing residues generated during ionic cross-linking, and carbonaceous char formed during polysaccharide degradation. Notably, L_GA_-MNP_10_ also exhibits relatively high residual mass and degradation events shifted toward higher temperatures, indicating that the alginate-rich matrix enhances thermal resistance. This behavior suggests that, even at lower NP loadings, polymer composition plays a decisive role in governing thermal stability, complementing the reinforcing effect of the magnetic phase.^26,27^

Density measurements further support the structural trends observed by thermal analysis. As summarized in Table S3, hydrogel density increases with NP loading, from 1.49 g·mL^–1^ for L_GA_-MNP_10_ to 1.66 g·mL^–1^ for H_GA_-MNP_20_. The higher density of H_GA_-MNP_20_ indicates a more compact internal structure resulting from the incorporation of the dense inorganic phase and more efficient packing of the polymeric network. Conversely, formulations with lower NP content exhibit slightly lower densities, consistent with higher free volume and greater chain mobility. These results align with previous reports, showing that metal oxide NPs enhance the structural integrity and mechanical stability of biopolymeric hydrogels.^28,29^

Overall, the combined FTIR, XRD, DSC, TGA, and density analyses demonstrate that GA-MNP are incorporated into the GA/SA network via coordination and ionic interactions, while preserving the magnetite crystalline structure. Increasing NP content leads to denser, more thermally stable ferrogels with constrained polymer mobility, highlighting the strong structure–property relationships that govern the performance of these 3D-printed magnetic hydrogels.

### 3.3 Swelling Behavior and pH Responsiveness

The swelling behavior (Table S4) of the hydrogels is governed by a complex interplay between polymer chemistry, Ca^2+^-mediated ionic cross-linking, and NP reinforcement, all of which are strongly influenced by the pH of the surrounding medium. The GA/SA network contains abundant hydroxyl (−OH) and carboxylic groups (−COOH/–COO^–^), whose ionization state directly controls intermolecular interactions, osmotic pressure, and network expansion. At acidic pH (pH 3), the carboxylate groups are predominantly protonated (−COO^–^ → −COOH), which reduces electrostatic repulsion between polymer chains. Simultaneously, protonation enhances intermolecular hydrogen bonding between −OH and −COOH groups, increasing water retention within the network. Under these conditions, swelling is driven not by electrostatic repulsion but by hydrogen-bonding-mediated hydration and the intrinsic hydrophilicity of the polysaccharide matrix, resulting in high capacity despite the reduced ionic contribution.^30^

At neutral pH (pH 7), partial ionization of carboxylic groups introduces moderate electrostatic repulsion; however, swelling becomes highly dependent on network integrity. In the NP-free hydrogel (HGA-Ctrl), Ca^2+^-mediated ionic cross-linking alone is insufficient to maintain structural stability, leading to network collapse and reduced swelling. In contrast, the incorporation of GA-MNP introduces additional physical cross-linking points, reinforcing the polymeric network, preventing structural collapse, and preserving swelling capacity^31^

At alkaline pH (pH 9), deprotonation of carboxylic groups (−COOH → −COO^–^) increases electrostatic repulsion between polymer chains, generating an osmotic driving force for water uptake and network expansion. However, this expansion is modulated by both the density of ionic cross-links and the rigidity introduced by NP incorporation. In highly loaded systems such as H_GA_-MNP_20_, the increased network compactness and reduced chain mobility limit excessive swelling, resulting in lower expansion compared to less densely cross-linked systems such as H_GA_-MNP_10_.^30^

Overall, the swelling response reflects a balance between hydrogen bonding, electrostatic interactions, Ca^2+^-mediated ionic cross-linking, and nanoparticle-induced reinforcement. This synergistic interplay enables tunable pH-responsive behavior, which is critical for optimizing mass transfer and adsorption performance in aqueous remediation systems.

### 3.4 Adsorptive Performance toward Imidacloprid

The adsorption profiles obtained at different initial imidacloprid concentrations (5–20 mg·L^–1^) (Figure 6) revealed clear differences in performance among the GA/SA hydrogels, confirming the strong influence of polymer composition and nanoparticle loading on sorption efficiency. Among the materials, H_GA_-MNP_20_ consistently exhibited the highest adsorption capacity, reaching 38.46 mg·g^–1^ at *C*_0_ = 20 mg·L^–1^, in agreement with the increase in active-site density expected from its higher nanoparticle content (20% v/v). Such enhancement is consistent with reports showing that hybrid materials enriched with magnetic or graphitic structures display greater electron density and a larger number of interaction sites, thereby improving affinity toward organic contaminants.^32^

**Figure 6:**
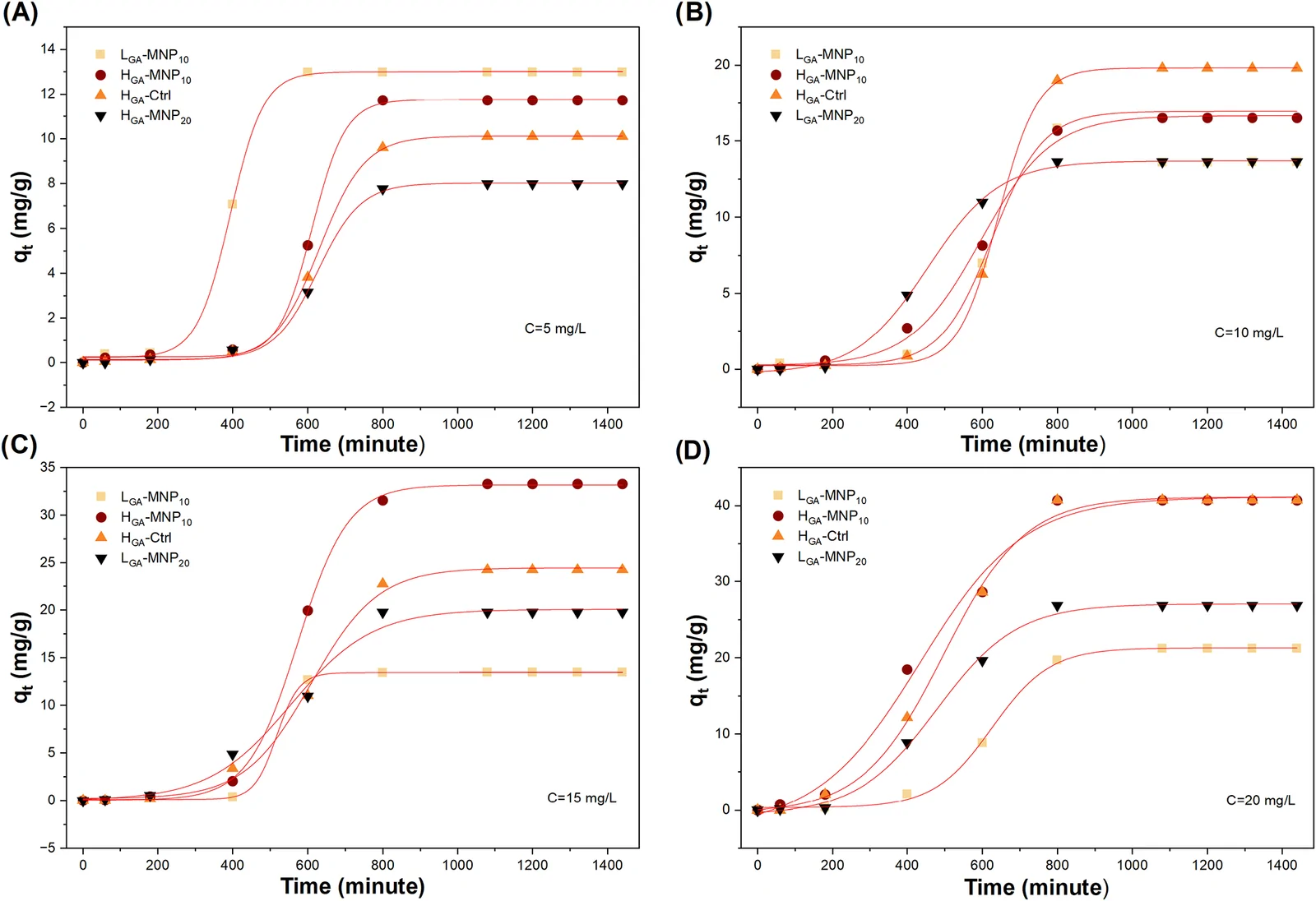
Adsorption kinetics of imidacloprid onto 3D-printed GA/SA-based hydrogels with different magnetic nanoparticle contents. Time-dependent adsorption profiles (*q*_t_) obtained at initial imidacloprid concentrations of (A) 5 mg L^–1^, (B) 10 mg L^–1^, (C) 15 mg L^–1^, and (D) 20 mg L^–1^. Symbols represent experimental data, while solid lines correspond to pseudo-second-order (PSO) kinetic model fittings. All formulations exhibit rapid initial uptake followed by diffusion-limited approach to equilibrium, with ferrogels containing higher GA-MNP loadings—particularly HGA-MNP_20_—showing increased adsorption capacity and a faster kinetic response. The excellent agreement between the experimental data and the PSO fits indicates that imidacloprid adsorption is governed by surface-controlled interactions involving functional groups from the GA/SA matrix and the magnetic nanoparticle domains.

Additionally, some adsorption kinetic profiles exhibited sigmoidal behavior, characterized by an initial rapid uptake, followed by a transient lag phase and a subsequent increase in adsorption before equilibrium was attained. Although such behavior is relatively uncommon in liquid-phase adsorption systems, it has been associated with complex adsorption mechanisms involving multistep mass-transfer processes, cooperative adsorption effects, or strong sorbate–sorbate interactions.^33^ In the present hydrogel system, however, the observed kinetic pattern is more likely related to the combined effects of adsorbate diffusion and matrix swelling. During the initial stage, imidacloprid molecules rapidly occupy the adsorption sites located on the external surface of the hydrogel. This is followed by a diffusion-controlled period, during which the transport of adsorbate molecules into the inner regions of the polymeric network becomes progressively slower. As hydration advances, the GA/SA matrix undergoes swelling and structural relaxation, leading to increased pore accessibility and the exposure of previously inaccessible adsorption sites within the hydrogel interior.^20,33^ The gradual activation of these internal sorption domains results in a second adsorption stage before equilibrium is reached. This interpretation is further supported by the intraparticle diffusion (Weber–Morris) model (Table S5), which provided relatively high correlation coefficients (*R* ^2^ = 0.93–0.95) for all hydrogel formulations, confirming the significant contribution of diffusion processes to imidacloprid uptake. However, the positive intercept values obtained (*C* = 0.94–7.76 mg·g^–1^) indicate that the diffusion plots did not pass through the origin, demonstrating that intraparticle diffusion is not the sole rate-controlling mechanism. Instead, adsorption is governed by a combination of external mass transfer, intraparticle diffusion, and surface adsorption phenomena.^34^ Furthermore, the Elovich model (Table S6) exhibited considerably lower correlation coefficients (*R* ^2^ = 0.65–0.67), suggesting that chemisorption on heterogeneous surfaces is not the dominant kinetic mechanism. Taken together, these findings support the hypothesis that the sigmoidal adsorption profiles arise primarily from a diffusion–swelling coupled process, in which hydration-induced expansion of the GA/SA network progressively exposes internal adsorption sites, generating the secondary adsorption stage observed before equilibrium is reached.^20,33^

A more detailed analysis of the adsorption behavior reveals that the performance differences between H_GA_-MNP_20_ and H_GA_-MNP_10_ arise from the combined effects of nanoparticle loading, matrix organization, and functional group accessibility, despite both formulations sharing the same GA/SA ratio. In H_GA_-MNP_10_, the relatively low nanoparticle content results in a polymer-dominated network, where the high density of hydrophilic functional groups (−OH and – COOH) promotes strong water–polymer interactions. This highly hydrated environment favors water retention within the matrix but simultaneously limits access to active sites for imidacloprid adsorption. In addition, the relatively compact and homogeneous structure restricts diffusion pathways, further reducing adsorption efficiency.^31^

In contrast, H_GA_-MNP_20_ exhibits a markedly different structural organization due to its higher nanoparticle loading. The increased presence of GA-MNP introduces additional adsorption sites through surface −OH groups and iron oxide domains, while also promoting the formation of a more heterogeneous network.^31^ This heterogeneity enhances surface roughness, increases interfacial area, and improves mass transfer within the hydrogel. Moreover, nanoparticle clusters act as localized domains that disrupt excessive polymer packing, increasing the accessibility of functional groups that would otherwise be partially shielded in a more compact matrix.

Thus, although both systems share the same polymer composition, the higher nanoparticle content in H_GA_-MNP_20_ shifts the balance from a hydration-dominated system toward a structure with greater interfacial complexity and active-site availability. This results in significantly enhanced adsorption capacity and faster kinetic response. L_GA_-MNP_10_, which contains a higher proportion of alginate, exhibited intermediate performance but matched the Langmuir model particularly well (*R* ^2^ = 0.99, Table S7), suggesting homogeneous monolayer adsorption dominated by electrostatic interactions between alginate carboxylate groups and imidacloprid.^33,35^ The nanoparticle-free hydrogel (H_GA_-Ctrl) presented intermediate adsorption capacity, reinforcing the synergistic role of magnetite domains in increasing available active sites and enhancing structural integrity.^36,37^

Kinetic analysis further supported these trends. Although all hydrogels exhibited the typical rapid uptake followed by a diffusion-limited plateau, the pseudo-second-order (PSO) model consistently provided the best fit, with R^2^ ≈ 0.99 for all materials and concentrations (Table S8). Beyond statistical agreement, the suitability of the PSO model is consistent with the adsorption mechanism, as it assumes that the rate-limiting step is governed by surface interactions involving active functional groups. In this system, hydroxyl (−OH) and carboxyl (−COO^–^/–COOH) groups from the GA/SA matrix, along with additional adsorption sites provided by GA-MNP, play a dominant role in imidacloprid binding, supporting a surface-controlled process with chemisorption-like contributions.

The adsorption profiles also suggest that mass transfer phenomena contribute to the overall process. The initial rapid uptake stage can be attributed to external mass transfer (boundary-layer diffusion), in which imidacloprid molecules quickly access readily available surface sites. This is followed by a slower approach to equilibrium, indicating the progressive diffusion of the adsorbate within the hydrogel network. However, the absence of distinct multilinear regions in the kinetic curves suggests that intraparticle diffusion is not the sole rate-limiting step, but rather occurs simultaneously with surface interactions. The inferior performance of the pseudo-first-order model (Table S9) further supports that physisorption alone cannot adequately describe the system. Altogether, these results indicate that the adsorption process is governed by a combination of diffusion phenomena and surface-controlled interactions, with the latter playing a dominant role.

Equilibrium modeling corroborated the kinetic findings. Langmuir fits indicated monolayer adsorption for the GA/SA-based hydrogels, with especially high correlation for L_GA_-MNP_10_ and H_GA_-MNP_10_ (*R* ^2^ ≥ 0.99), while H_GA_-MNP_20_ achieved the highest monolayer adsorption capacity (*q*_m_ = 38.02 mg·g^–1^, Table S7). The combination of increased magnetic nanosites and the structural organization within the GA/SA network explains this marked enhancement, consistent with studies demonstrating the role of magnetite nanodomains in expanding surface heterogeneity and active-site richness.^36,37^ The more moderate performance of H_GA_-MNP_10_ and L_GA_-MNP_10_ reflects the balance between hydrophilicity, polymer packing, and the distribution of carboxylate-rich domains within the alginate matrix.

Altogether, the adsorption mechanism can be described as monolayer adsorption on a heterogeneous surface, governed by hydrogen bonding, electrostatic interactions, and nanoparticle-mediated active sites. Such behavior aligns with that observed in other advanced biobased magnetic systems.^37^ Among all evaluated formulations, H_GA_-MNP_20_ demonstrated the most favorable combination of adsorption capacity, kinetic performance, and structural stability, further benefiting from magnetic retrievability, making it a highly promising material for neonicotinoid removal in water treatment applications.

The adsorption mechanism of imidacloprid onto 3D-printed hydrogels based on GA and SA is primarily governed by H-bonding (hydrogen bonding), electrostatic interactions, and surface complexation. The hydroxyl (−OH) and carboxyl (−COOH/–COO^–^) groups of GA and SA act as hydrogen-bond donors and acceptors, forming strong H-bonding interactions with the electronegative nitrogen and oxygen atoms of imidacloprid, thereby enhancing molecular affinity and stabilization within the polymeric network.^35^ Additionally, the ionized carboxylate groups of alginate promote electrostatic interactions with positively charged regions of the pesticide, particularly at pH values that favor surface charge interactions.^38^ The incorporation of Fe_3_O_4_ NP introduces extra active sites via surface – OH groups, enabling additional H-bonding and coordination/surface complexation mechanisms, while also increasing surface area and allowing magnetic recovery of the adsorbent. Finally, the GA/SA ratio modulates porosity, functional group density, and diffusion pathways, directly influencing adsorption capacity and kinetics.^35^

Postadsorption FTIR spectra were also analyzed to identify possible interactions between IMD and the adsorbent surface. However, no significant shifts or new absorption bands were observed after adsorption. This behavior may be attributed to the predominance of weak noncovalent interactions and to the drying step required for FTIR sample preparation, which can disrupt or mask interactions occurring at the solid–liquid interface during adsorption.

### 3.5 Reusability and Regeneration Performance

The reusability of adsorbents is a key parameter for evaluating their applicability in water treatment. The 3D-printed GA/SA and GA-MNP hydrogels showed good regeneration performance during successive adsorption–desorption cycles of imidacloprid (Table S10). When the hydrogels were only superficially rinsed with distilled water (without desorbing solution), the adsorption capacity was maintained for two cycles. In contrast, regeneration with 70% ethanol was performed up to four cycles, yielding equilibrium adsorption capacities of 33.28 mg g^–1^ (*R* = 100%) and 33.02 mg g^–1^ (*R* = 99%) for cycles 1 and 2, respectively. A gradual decrease was observed in subsequent cycles, reaching 21.22 mg g^–1^ (*R* = 64%) in cycle 3 and 11.35 mg g^–1^ (*R* = 35%) in cycle 4. Conversely, regeneration with KCl (0.5 M) led to hydrogel degradation, likely due to ionic exchange between K^+^ and Ca^2+^ ions, which disrupts the Ca^2+^-alginate cross-linking (“egg-box” structure) and weakens the polymer network.^36^ These results highlight the importance of selecting an appropriate desorption agent to preserve hydrogel stability and reusability.

The reusability results also provide important insights into the structural stability of the hydrogels during repeated adsorption–desorption cycles. Under ethanol regeneration, the hydrogels maintained high adsorption capacity during the initial cycles, followed by a gradual decrease in performance. This behavior can be attributed to partial structural rearrangement of the polymeric network, as well as progressive modification or partial blocking of active adsorption sites.^37^

Ethanol, while effective as a desorption agent by disrupting hydrogen bonding and solubilizing the adsorbed pesticide, may also induce partial dehydration of the hydrogel matrix. This can lead to slight contraction of the network, affecting porosity and diffusion pathways without causing complete structural collapse.^31^ As a result, although the overall integrity of the hydrogel is preserved, subtle changes in morphology and internal organization may occur over successive cycles.

In contrast, the use of KCl (0.5 M) leads to significant structural degradation due to ionic exchange between K^+^ and Ca^2+^ ions, disrupting the Ca^2+^-mediated “egg-box” cross-linking of alginate and resulting in a loss of network stability. Regarding magnetic properties, the Fe_3_O_4_ nanoparticles are expected to retain their magnetic response throughout the regeneration cycles due to the chemical stability of the magnetite phase. However, minor redistribution or aggregation of nanoparticles within the matrix cannot be excluded, which may slightly influence local adsorption behavior.

Overall, the adsorption mechanism, primarily governed by hydrogen bonding, electrostatic interactions, and surface complexation, is preserved after reuse. However, the gradual decrease in adsorption capacity suggests that repeated cycles affect the accessibility and distribution of active sites, rather than fundamentally altering the adsorption mechanism.

Compared with previously reported adsorbents for imidacloprid removal, the proposed 3D-printed GA/SA-MNP hydrogels exhibit competitive adsorption performance while offering additional functional advantages, as summarized in Table S11. Although its adsorption capacity is lower than that of high-surface-area materials such as activated carbon/magnetite composites, the present system offers a unique combination of properties not simultaneously achieved by the other adsorbents.

Unlike biochar or microplastic-based systems, the GA/SA-MNP hydrogels enable straightforward magnetic separation and demonstrate improved reusability. Moreover, the incorporation of 3D-printed architecture enables precise structural tunability, enhancing mass transfer and the accessibility of active sites, thereby contributing to efficient adsorption performance. Overall, the proposed system represents a balanced approach that prioritizes structural control, recoverability, and multifunctionality, while maintaining competitive adsorption efficiency.

Although adsorption capacity progressively decreased during successive regeneration cycles, the magnetic hydrogels retained sufficient magnetic responsiveness to enable efficient recovery by external magnetic separation after every cycle, including the fourth cycle. While quantitative magnetic measurements were not performed after regeneration, the successful magnetic separation observed throughout the experiments indicates that the Fe_3_O_4_ nanoparticles remained effectively incorporated within the hydrogel matrix.

## 4 Conclusion

The integration of GA, SA, and GA-MNP enabled the fabrication of 3D-printed ferrogels with tunable surface morphology, density, thermal stability, and pH-responsive swelling behavior. The incorporation of MNP acted as an effective structuring and reinforcing agent, improving network integrity, preventing hydrogel collapse under neutral conditions, and enhancing thermal resistance. Swelling studies confirmed a pronounced pH responsiveness, with maximum hydration under acidic conditions and controlled re-expansion at alkaline pH, demonstrating the potential of these materials for stimuli-responsive applications. Adsorption experiments further revealed that both polymer composition and nanoparticle loading critically govern imidacloprid removal. Among all formulations, H_GA_-MNP_20_ exhibited the highest adsorption capacity (38.46 mg·g^–1^), faster kinetics, and superior structural stability. The adsorption process followed pseudo-second-order kinetics and was best described by the Langmuir isotherm, indicating a surface-controlled, monolayer mechanism mediated by hydrogen bonding, electrostatic interactions, and nanoparticle-derived active sites.

Overall, this work demonstrates that combining natural polysaccharides, magnetic nanostructures, and additive manufacturing constitutes a robust and sustainable strategy for water remediation. The 3D-printed GA/SA magnetic hydrogels combine high adsorption efficiency, tunable architecture, and magnetic retrievability, making them promising candidates for treating pesticide-contaminated waters and for the development of reusable, multifunctional remediation systems. Beyond the specific case of imidacloprid removal, the present approach highlights the potential of combining natural polysaccharides, MNP, and additive manufacturing to design customizable remediation platforms with tunable architecture and functionality. Future studies should focus on improving long-term regeneration performance, evaluating the adsorption of broader classes of emerging contaminants, and assessing the behavior of these materials under more complex environmental conditions. Such advances may contribute to the development of scalable and sustainable technologies for water treatment and environmental remediation.

## Supplementary Material



## Notes

Ink compositions (Table S1); FTIR band assignments and representative band shifts (Table S2); density measurements (Table S3); swelling behavior (Table S4); intraparticle diffusion (Weber–Morris) model parameters (Table S5); Elovich model parameters (Table S6); adsorption isotherm parameters (Table S7); pseudo-second-order kinetic parameters (Table S8); pseudo-first-order kinetic parameters (Table S9); regeneration and reusability data (Table S10); comparison with previously reported adsorbents (Table S11); TEM images (Figure S1); and AFM images (Figure S2) (PDF)
