# Supplementary material for: 3D-Printed Magnetic Polysaccharide Hydrogels as Sustainable Platforms for Pesticide Remediation
Source: ACS Omega. 2026 Aug 7;11(32):47866–78. doi: 10.1021/acsomega.6c03353 (PMC13491463; doi:10.1021/acsomega.6c03353)
Supplement: Supplementary file 1 [file ao6c03353_si_001.pdf]

SUPPORTING INFORMATION

3D-Printed Magnetic Polysaccharide Hydrogels as Sustainable Platforms for Pesticide Remediation

Yulianis P. Barragán Medina[1,2]. Jéssica S. Rodrigues[2]. Amanda S. M de Freitas[2]. Gabriela P. U. Villarreal[2]. Jimena S. Gonzalez[1]*. Leonardo F. Fraceto[2]*

[1] Grupo de Materiales Compuestos Termoplásticos. Instituto de Investigaciones en Ciencia y Tecnología de Materiales (INTEMA). CONICET-UNMdP. Av. Colón 10850. 7600. Mar del Plata. BA. Argentina.

[2] Institute of Science and Technology. São Paulo State University (UNESP). Av. Três de Março 511. 18087-180. Sorocaba. SP. Brazil.

*Corresponding author: leonardo.fraceto@unesp.br; jimena.s.gonzalez@gmail.com

Table S1. Composition of 3D-printable biopolymeric inks, including GA/SA ratios, polymer concentrations, and GA-MNP contents.

Ink	Description	GA/SA (w/w)	GA (% w/v)	SA (% w/v)	GA-MNP aqueous dispersion at 4 wt% (% v/v)	GA-MNP solids (% w/w)
L_{GA}-MNP_{10}	Low GA / High SA. 10% v/v GA-MNP	0.04	1.8	45.0	10.0	0.4
H_{GA}-MNP_{10}	High GA / Low SA. 10% v/v GA-MNP	0.50	9.0	18.0	10.0	0.4
H_{GA}-Ctrl	High GA / Low SA. without GA-MNP	0.50	10.0	20.0	0.0	0.0
H_{GA}-MNP_{20}	High GA / Low SA. 20% v/v GA-MNP	0.50	8.0	16.0	20.0	0.8

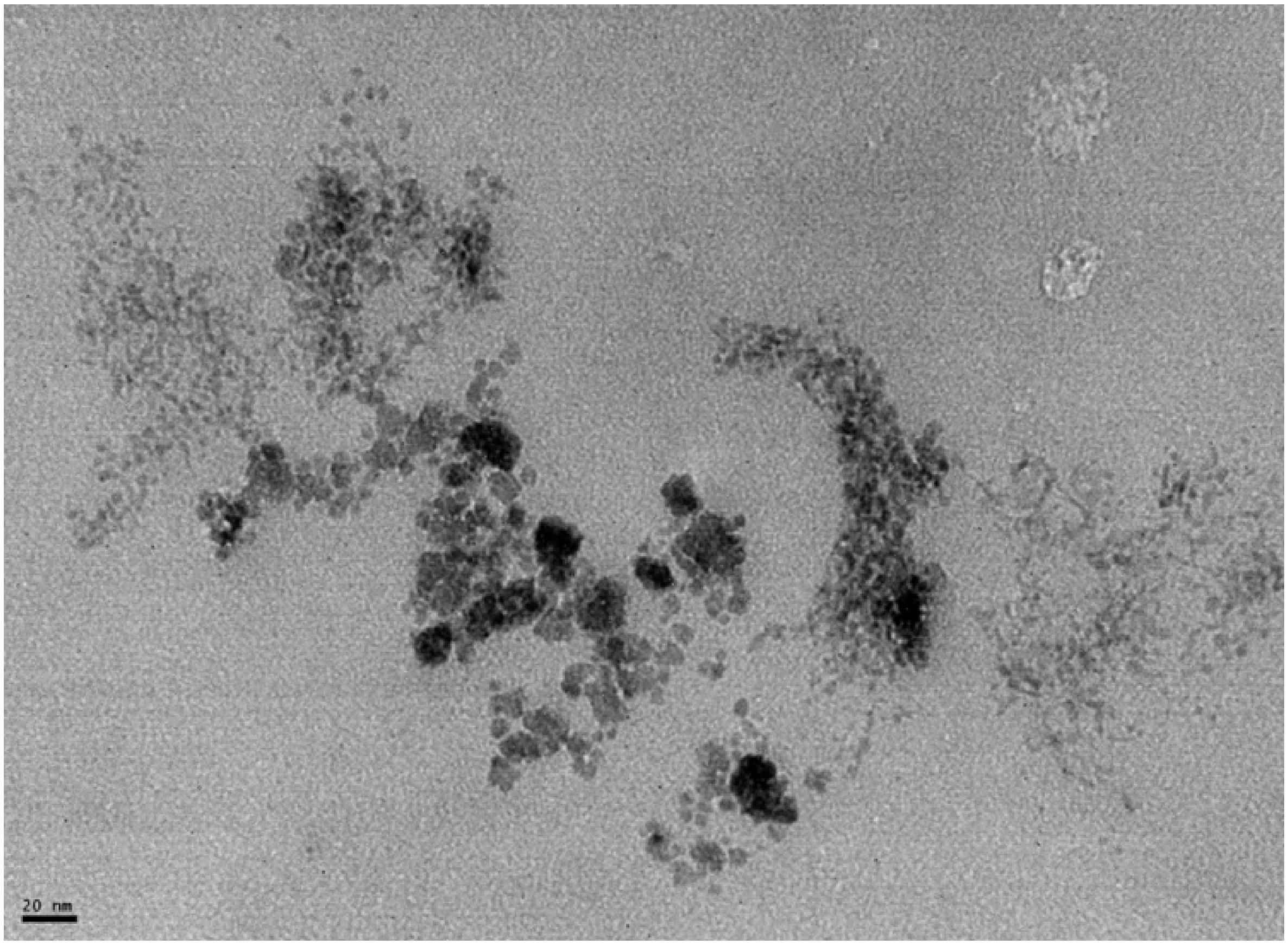

Figure S1. Transmission electron microscopy (TEM) micrograph of gum arabic–stabilized magnetite nanoparticles (GA-MNP). The image reveals nearly spherical nanometric magnetite nanoparticles forming small clusters, consistent with magnetic dipole–dipole interactions. The observed particle size and morphology confirm the formation of well-defined nanocrystalline domains stabilized by the gum arabic coating, in agreement with the XRD and NTA analyses.

Table S2. Main FTIR band assignments and representative band shifts observed between GA-MNP and the 3D-printed GA/SA ferrogels.

GA-MNP (cm^{-1})	Printed ferrogels (cm^{-1})	Band Shift, Δν (cm^{-1})	Assignment	Interpretation	References
3700	3686	-64	O–H stretching	Broad band associated with hydrogen bonding within the polysaccharide network	[1]
2996	2970	−26	C–H stretching	Minor shift associated with incorporation into the GA/SA hydrogel matrix	[1]
1502	1498	−4	COO^- / C=O-related vibration	Suggests interaction between oxygenated groups and Fe_3O_4/Ca^{2+} crosslinked network	[2]
1367	1341	−26	COO^- / C–O vibration	Indicates changes in the local environment of carboxylate groups after ionic crosslinking	[2]
1222	1215	−7	C–O–C / C–O stretching	Consistent with polymer–nanoparticle interactions within the hydrogel network	[3]
793	786	−7	Fe–O stretching	Confirms incorporation of magnetite domains and suggests interaction with the polysaccharide matrix	[4]

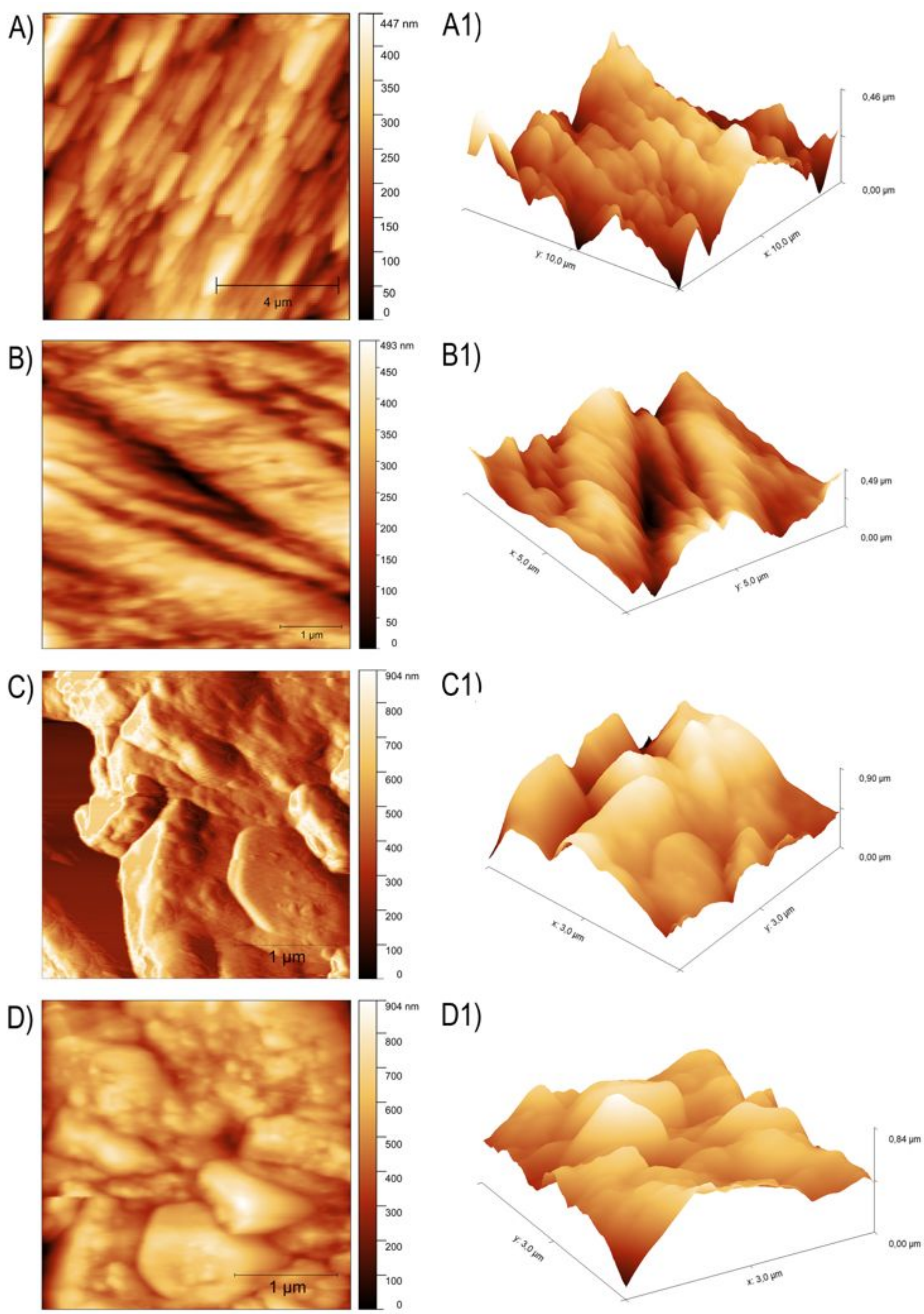

Figure S2. Representative 2D height images (left) and corresponding 3D topographical reconstructions (right) of (A, A1) LGA-MNP10, (B, B1) HGA-MNP10, (C, C1) HGA-Ctrl, and (D, D1) HGA-MNP20. The images reveal formulation-dependent surface organization, with nanoparticle-containing ferrogels exhibiting more structured and compact topographies than the nanoparticle-free control. Increasing GA-MNP loading enhances surface organization and reduces roughness heterogeneity, indicating stronger polymer–nanoparticle interactions and improved network cohesion within the printed hydrogel matrices.

Table S3. Density values of 3D-printed GA/SA hydrogels with different GA-MNP contents. determined by helium pycnometry (mean ± standard deviation).

Gels	L_{GA}-MNP_{10}	H_{GA}-MNP_{10}	H_{GA}-Ctrl	H_{GA}-MNP_{20}
**Density (g/mL)**	1.49 ± 0.1	1.56 ± 0.1	1.58 ± 0.1	1.66 ± 0.1

Table S4. Swelling measurements at pH 3, 7, and 9, determined at room temperature (23 °C) (mean ± standard deviation).

**Sample**	**Swelling Degree (%)**		
	**pH=3**	**pH=7**	**pH=9**
L_{GA}-MNP_{10}	213 ± 14	161 ± 7	116 ± 13
H_{GA}-MNP_{10}	254 ± 14	183 ± 7	235 ± 15
H_{GA}-Ctrl	213 ± 6	5 ± 0	177 ± 18
H_{GA}-MNP_{20}	166 ± 17	181 ± 6	171 ± 15

Table S5. Weber–Morris intraparticle diffusion model parameters for Imidacloprid adsorption onto GA/SA 3D-printed hydrogels

Gel	Co (mg/L)	Kp	C	R^2
L_{GA}-MNP_{10}	5	0.418	2.201	0.94
	10	0.548	2.968	0.94
	15	0.418	2.201	0.94
	20	0.418	2.201	0.94
H_{GA}-MNP_{10}	5	0.379	2.046	0.94
	10	0.536	0.943	0.94
	15	1.862	6.192	0.93
	20	1.183	1.136	0.95
L_{GA}-Ctrl	5	0.329	1.868	0.93
	10	0.647	3.664	0.93
	15	0.793	4.568	0.93
	20	1.334	7.763	0.93
H_{GA}-MNP_{20}	5	0.261	1.481	0.94
	10	0.445	2.550	0.93
	15	0.643	3.570	0.94
	20	0.876	4.969	0.93

Table S6. Elovich Model parameter for Imidacloprid adsorption onto GA/SA 3D-printed hydrogels

Gel	Co (mg/L)	k_e (g/mg·min$^{-1)}$	A	R^2
L_{GA}-MNP_{10}	5	2,077	3,691	0,67
	10	2,738	4,880	0,66
	15	2,077	3,691	0,67
	20	2,077	3,691	0,67
H_{GA}-MNP_{10}	5	2,077	3,691	0,67
	10	2,714	4,880	0,66
	15	2,077	3,691	0,67
	20	2,077	3,691	0,67
L_{GA}-Ctrl	5	1,626	2,983	0,66
	10	3,185	5,847	0,66
	15	3,907	7,220	0,65
	20	6,563	12,176	0,65
H_{GA}-MNP_{20}	5	1,288	2,366	0,66
	10	2,196	4,049	0,66
	15	3,183	5,792	0,66
	20	4,322	7,930	0,66

Table S7. Langmuir and Freundlich isotherm model parameters for imidacloprid adsorption

Gel	q_m (mg/g)	K_L (L/mg)	R_2 (Langmuir)	n	K_F (L/mg)	R^2 (Freundlich)
L_{GA}-MNP_{10}	20.96	34940.6	0.99	40.65	17.15	0.08
H_{GA}-MNP_{10}	10.92	21834.06	1.0	2.64	2.02	0.53
H_{GA}-Ctrl	16.75	41876.05	0.67	5.61	9.66	0.52
H_{GA}-MNP_{20}	38.02	337.98	0.71	1.94	8.63	0.72

Table S8. Pseudo-second-order (PSO) kinetic parameters for imidacloprid adsorption onto GA/SA hydrogels

**Gel**	**C_0 (mg/L)**	**k_2 (g/mg·min)**	**q_2 (mg/g)**	**h (mg/g.min^{-1})**	**R^2**
L_{GA}-MNP_{10}	5	0.0011	12.25	0.1651	0.99
	10	0.0009	16.0	0.2304	0.99
	15	0.0013	12.71	0.2100	0.99
	20	0.0007	20.04	0.2811	0.99
H_{GA}-MNP_{10}	5	0.0012	11.07	0.1471	0.99
	10	0.0009	15.6	0.2190	0.99
	15	0.0004	31.45	0.3956	0.99
	20	0.0004	38.46	0.5917	0.99
H_{GA}-Ctrl	5	0.0014	11.07	0.1716	0.99
	10	0.0007	18.69	0.2445	0.99
	15	0.0006	22.94	0.3157	0.99
	20	0.0004	38.46	0.5917	0.99
H_{GA}-MNP20	5	0.0016	8.1	0.1050	0.99
	10	0.001	12.89	0.1662	0.99
	15	0.0007	18.69	0.2445	0.99
	20	0.0005	25.38	0.3221	0.99

Table S9. Pseudo-first-order (PFO) kinetic parameters for imidacloprid adsorption onto GA/SA hydrogels

Gel	C_0 (mg/L)	k_1 (g/mg·min^{-1})	q_1 (mg/g)	R^2
**L_{GA}-MNP$_{10}$**	5	1528.0	8.6	0.8
	10	1089.9	6.1	0.75
	15	951.0	0.5	0.94
	20	5783.1	17.3	0.94
**H_{GA}-MNP$_{10}$**	5	9441.9	31.3	0.93
	10	11.4	1.6	0.97
	15	976.2	0.7	0.95
	20	1086.5	14.2	0.94
**H_{GA}-Ctrl**	5	2099.5	1.6	1.0
	10	2498.1	3.8	0.99
	15	71.9	1.6	0.02
	20	68.3	53.8	0.34
**H_{GA}-MNP20**	5	62.9	1.2	0.02
	10	68.1	1.4	0.02
	15	1205.6	1.9	0.98
	20	14113.6	36.8	0.94

Table S10. Recycle use assays results for **H_{GA}-MNP_{10}**

Cycles	q_e (mg/g); C_0:15 mg/L	R%
Cycle 1	33.27	100%
Cycle 2	33.02	99%
Cycle 3	21.05	63%
Cycle 4	11.55	35%

Table S11. Comparative performance of different adsorbents for imidacloprid removal

Material	q_e (mg/g)	Magnetic separation	Reuse	3d Architecture	References
Activated carbon/magnetite	192	Yes	8 cycles	Large porous particle with nanoparticles attached to the surface	[5]
Biochar from eucalyptus wood chips	14.75	No	not specified	Rigid tubular structures inherited from wood	[6]
Aged microplastics (PE)	0.328	No	not specified	Irregular surface with folds. cracks. and layers	[7]
Alginate/arabic gum/iron oxides hydrogel.	33.28	Yes	4 cycles	Print gels 3D	Present work

REFERENCES

[1] Yunus AI, Chen Y, Bozeman JF, Darko SA, Darko SA, Bozeman JF. Facile fabrication of magnetite (Fe3O4) nanoparticles by hydrothermal carbonization of waste iron supplements. RSC Sustainability 2025;3:3530–47. https://doi.org/10.1039/d5su00312a.

[2] D'Elía NL, Rial Silva R, Sartuqui J, Ercoli D, Ruso J, Messina P, et al. Development and characterisation of bilayered periosteum-inspired composite membranes based on sodium alginate-hydroxyapatite nanoparticles. J Colloid Interface Sci 2020;572:408–20. https://doi.org/10.1016/j.jcis.2020.03.086.

[3] Feraru A, Tóth ZR, Mureșan-Pop M, Baia M, Gyulavári T, Páll E, et al. Anionic Polysaccharide Cryogels: Interaction and In Vitro Behavior of Alginate–Gum Arabic Composites. Polymers (Basel) 2023;15. https://doi.org/10.3390/polym15081844.

[4] Uddin MN, Alam S, Das H. Synthesis and optimization of cubic shaped magnetite nanoparticles by one-step ultrasound irradiation process. Solid State Commun 2025;396. https://doi.org/10.1016/j.ssc.2024.115772.

[5] Safe YL, Springer V, Avena M. A sustainable and fast methodology based on magnetic activated carbon for removal of imidacloprid from aqueous solution. J Environ Chem Eng 2023;11:111135. https://doi.org/10.1016/j.jece.2023.111135.

[6] Srikhaow A, Chaengsawang W, Kiatsiriroat T, Kajitvichyanukul P, Smith SM. Adsorption Kinetics of Imidacloprid, Acetamiprid and Methomyl Pesticides in Aqueous Solution onto Eucalyptus Woodchip Derived Biochar. Minerals 2022;12:528. https://doi.org/10.3390/min12050528.

[7] Wu H, Yuan Q, Chen B, Kong Q, Huang S, Cheng L, et al. Adsorption behaviors of atrazine and imidacloprid on high temperature aged microplastics: Mechanism and influencing factors. Desalination Water Treat 2025;321:100931. https://doi.org/10.1016/j.dwt.2024.100931.
